# Treatment of Catatonia Associated with Xp11.22 Duplication Syndrome and Comorbid Autism Spectrum Disorder: A Case Report

**DOI:** 10.1192/j.eurpsy.2025.1133

**Published:** 2025-08-26

**Authors:** C. J. Gideon, V. Lindegaard

**Affiliations:** 1 Michigan State University/Pine Rest, Grand Rapids, United States

## Abstract

**Introduction:**

Xp11.22 duplication syndrome, a rare genetic condition first identified in 2009, has fewer than 100 documented cases in the literature. To date, little is known about the genotype-phenotype relationship in this rare genetic syndrome and there is a paucity of data specifically regarding catatonia in this condition.

**Objectives:**

The primary aim of this case report is to provide a comprehensive description of the clinical presentation, diagnostic approach, and management strategies employed in a case of catatonia occurring in the context of Xp11.22 duplication syndrome and ASD.

**Methods:**

Methods of this case report include assessment of the patient via thorough psychiatric and medical evaluation, as well as additional information obtained through chart review and collateral sources.

**Results:**

Our patient, a 15-year-old Caucasian male, was diagnosed with Xp11.22 duplication syndrome at age 3 due to speech and motor delays. At 14, he experienced sudden behavioral and motor changes, including withdrawal, repetitive speech, slowed gait, and repetitive motor behaviors like “rewind” actions. Over 3 months, symptoms included emotional lability, self-injurious behaviors, and significant disruption to daily functioning. Outpatient management with olanzapine and quetiapine worsened agitation. Referred to a specialist, he was started on lorazepam up to 12 mg daily for suspected catatonia. ECT was considered, but further workup ruled out seizure disorder and autoimmune encephalitis. Pediatric neurology found no alternative etiology and recommended ECT. On admission, BFCRS score was 14, showing catatonic symptoms like automatic obedience, mutism, and immobility. Lorazepam was reduced to 2 mg three times daily, and amantadine 100 mg twice daily was continued. An acute ECT course of 12 bilateral treatments over four weeks reduced catatonic symptoms, improving mutism, motor speed, and daily activities. During a 56-day hospitalization, he received 7 maintenance treatments. Upon discharge, BFCRS decreased to 7, and he continued lorazepam and amantadine.

**Conclusions:**

This case report underscores the complexity of managing catatonia in patients with Xp11.22 duplication syndrome and ASD, highlighting the potential need for multimodal treatment approaches. The significant improvement observed with the addition of ECT to the treatment regimen emphasizes the importance of considering this option in cases of refractory catatonia, even in patients with complex genetic and neurodevelopmental backgrounds. This case raises important questions about the underlying neurobiological mechanisms of catatonia in the context of Xp11.22 duplication syndrome and ASD.

**Disclosure of Interest:**

None Declared

